# Computer-Simulated Biopsy Marking System for Endoscopic Surveillance of Gastric Lesions: A Pilot Study

**DOI:** 10.1155/2015/197270

**Published:** 2015-04-14

**Authors:** Weiling Hu, Bin Wang, Leimin Sun, Shujie Chen, Liangjing Wang, Kan Wang, Jiaguo Wu, John J. Kim, Jiquan Liu, Ning Dai, Huilong Duan, Jianmin Si

**Affiliations:** ^1^Department of Gastroenterology Sir Run Run Shaw Hospital, Zhejiang University, Hangzhou 310016, China; ^2^Institute of Gastroenterology, Zhejiang University, Hangzhou 310016, China; ^3^College of Biomedical Engineering & Instrument Science, Zhejiang University, Hangzhou 310027, China; ^4^Key Laboratory of Biomedical Engineering, Ministry of Education, Zhejiang University, Hangzhou 310027, China; ^5^Division of Gastroenterology, Loma Linda University, Loma Linda, CA 92354, USA

## Abstract

Endoscopic tattoo with India ink injection for surveillance of premalignant gastric lesions is technically cumbersome and may not be durable. The aim of the study is to evaluate the accuracy of a novel, computer-simulated biopsy marking system (CSBMS) developed for the endoscopic marking of gastric lesions. Twenty-five patients with history of gastric intestinal metaplasia received both CSBMS-guided marking and India ink injection in five points in the stomach at index endoscopy. A second endoscopy was performed at three months. Primary outcome was accuracy of CSBMS (distance between CSBMS probe-guided site and tattoo site measured by CSBMS). The mean accuracy of CSBMS at angularis was 5.3 ± 2.2 mm, antral lesser curvature 5.7 ± 1.4 mm, antral greater curvature 6.1 ± 1.1 mm, antral anterior wall 6.9 ± 1.6 mm, and antral posterior wall 6.9 ± 1.6 mm. CSBMS (2.3 ± 0.9 versus 12.5 ± 4.6 seconds; *P* = 0.02) required less procedure time compared to endoscopic tattooing. No adverse events were encountered. CSBMS accurately identified previously marked gastric sites by endoscopic tattooing within 1 cm on follow-up endoscopy.

## 1. Introduction

Gastric cancer is the second most common cause of cancer-related death worldwide [1, 2], and endoscopic surveillance of premalignant lesions may improve outcome [3, 4]. When polypoid or flat mucosal lesions are encountered during upper endoscopy, India ink is commonly injected near the lesion to facilitate endoscopic surveillance. However, injection of India ink is procedurally cumbersome with a risk of technical failure [5]. Furthermore, endoscopic tattoo may fade over time and limit long-term follow-up.

We have previously introduced image-guided biopsy marking system (IGBMS) that utilizes a computer-generated, electromagnetic tracking device to identify previously marked sites during upper endoscopy [6]. Since our initial work, we have changed the calculation method to improve the accuracy. We performed a pilot study to evaluate the effectiveness of computer-simulated biopsy marking system (CSBMS) developed for surveillance of premalignant gastric lesions.

## 2. Method

The study protocol conforms to the ethical guidelines of the 1975 Declaration of Helsinki (6th revision, 2008) and was approved by the ethics committee of Sir Run Run Shaw Hospital prior to initiating this study. Twenty-five patients with history of gastric intestinal metaplasia [7], who were scheduled for surveillance endoscopy according to society guidelines [8, 9], were prospectively enrolled between October 2012 and April 2014 at Sir Run Run Shaw Hospital. Informed consent was obtained from each patient prior to enrollment.

### 2.1. CSBMS Components

**Figure 1:**
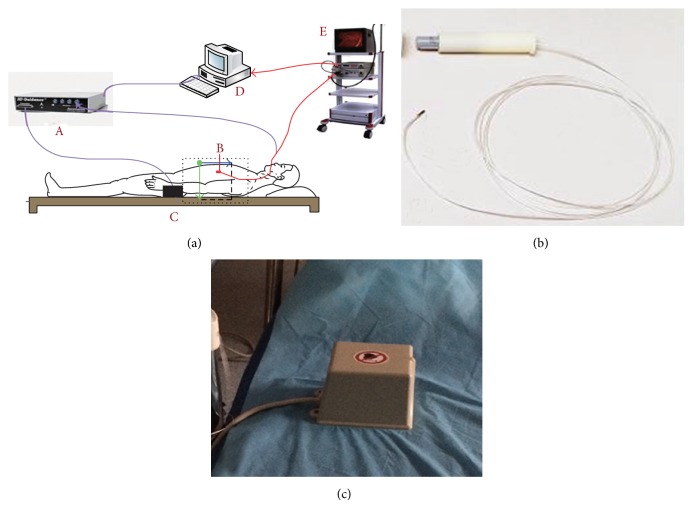
(a) Schematic diagram of the CSMBS components: A electronics unit device, B probe and endoscope together in the stomach, C transmitter, D imaging workstation, E gastroscope system. (b) Probe. (c) Transmitter. Figures 1(a) and 1(b) [6] were included for reader's convenience.

The components of the CSBMS include electronic unit device, sensor probe, magnetic transmitter, and CSBMS workstation (Figure 1(a)). Electronic unit device, sensor probe, and magnetic transmitter (Ascension Technology Corp, Burlington, Vermont, USA) are integrated as an electromagnetic tracking system that provides real-time information on the position of the endoscope tip during the procedure. The probe (Figure 1(b)) is 1.3 mm in diameter and 6.5 mm in length. The cable is 0.6 mm in diameter and 1.8 m in length. The probe is held adjacent to the tip of the endoscope and can provide the probe position using magnetic coordinates. The magnetic transmitter's effective position region is 50 cm × 50 cm × 50 cm. The magnetic transmitter is placed near the patient's abdomen to ensure that the probe is within the magnetic region during the procedure (Figure 1(c)).

### 2.2. Methods and Outcomes

A conventional upper endoscope (GIF-QX240 EGD, Olympus Corp., Japan) was used for all procedures. Although all patients were recommended to receive moderate sedation (fentanyl and midazolam), three patients refused to receive any sedation for the index and follow-up procedures. Prior to the insertion of the endoscope, the CSBMS probe was held adjacent to the scope at the 12 o'clock position. Both CSBMS and endoscopy images were continuously recorded. With the hand-eye calibration method [10], the probe recorded the position of the scope using magnetic field coordinates. After the stomach was intubated, five reference points were touched with the tip of the endoscope (cardia, junction of the anterior body wall and angularis, junction of the posterior body wall and angularis, middle of the angularis, and pylorus). Afterwards, five arbitrary points were selected in the antrum (lesser curvature of the antrum, greater curvature of the antrum, angularis, anterior wall, and posterior wall of the antrum). According to our previous tattooing technique [11], India ink was injected and a biopsy was performed directly over the site [12]. Finally, the center of the biopsy site was touched with the tip of the endoscope and was designated as the CSBMS marking point. The procedure times for tattoo injection and CSBMS were recorded. Afterwards, three-dimensional virtual model of the stomach including five biopsy points was constructed.

**Figure 2:**
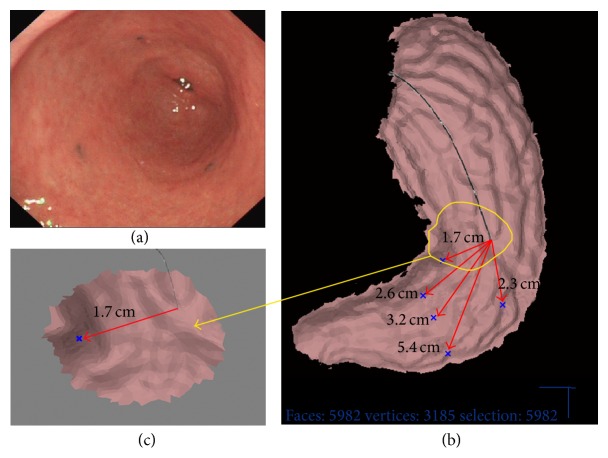
The endoscope is navigated by the CSMBS. (a) Live endoscopy image. (b) Reconstructed gastric model. The blue markers are the simulated CTBMS marked sites. (c) CSBMS directs the endoscope to the nearest marked site.

**Figure 3:**
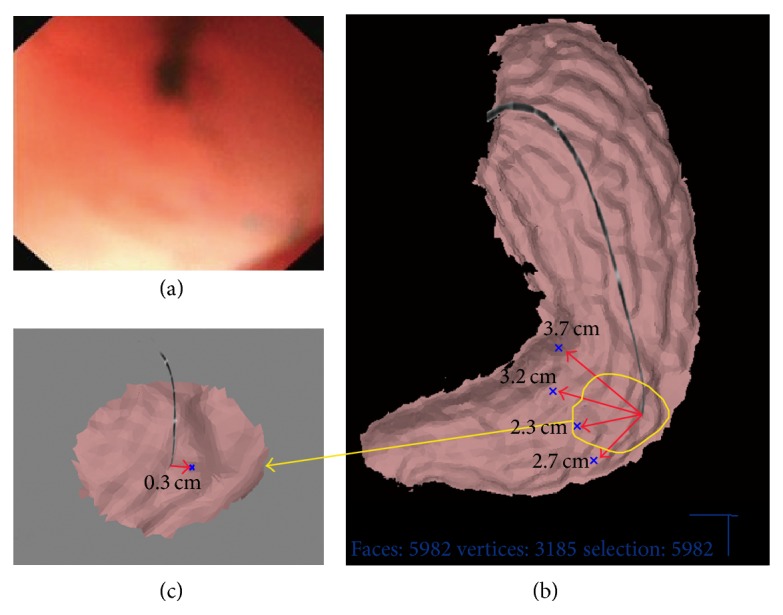
The previously marked points are reached by the CSBMS at follow-up endoscopy. (a) Live endoscopy image showing the scope touching the mucosa previously marked with India ink. (b) Reconstructed gastric model. The distance between the endoscope tip and the blue marker decreases as the CSBMS probe approaches the previously marked site (blue marker). (c) Measurement of CSBMS accuracy using CSBMS.

At three months, a follow-up endoscopy was performed with CSBMS. The five reference points were touched by the endoscope. Based on the reference point positions between the first and second procedures, CSBMS marking points from the index procedure were transferred to the second gastric model (Figure 2(a)). Subsequently, CSBMS directed the endoscope to the previously designated CSBMS marking points (Figure 3(a)). Primary outcome of the study was the accuracy of CSBMS (distance between CSBMS probe-guided site and tattoo site measured by CSBMS). Secondary outcome was procedure time.

### 2.3. Definitions and Statistics

The accuracy was defined by the distance between the CSBMS marking point and the center of the tattoo, measured by CSBMS (Figure 3(b)). Prior to the measurement, the distance in the stomach was calibrated using the size on an open biopsy forceps of 6 mm. CSBMS marking time was defined by the time required to identify and touch every tattoo point by the probe recorded by the CSBMS. India ink marking time was defined by the time required to inject India ink to create a submucosa tattoo. Accuracy was summarized by means and standard deviation. Student's* t*-test was used to compare CSBMS and India ink marking time.

## 3. Experimental Results

All 25 patients had moderate to severe intestinal metaplasia in one or more gastric sites during the index endoscopy (Table 1). During follow-up endoscopy, one of five tattoo points was not visible in two (8%) patients due to fading. The mean accuracy at angularis was 5.3 ± 2.2 mm, antral lesser curvature 5.7 ± 1.4 mm, antral greater curvature 6.1 ± 1.1 mm, anterior body 6.9 ± 1.6 mm, and posterior body 6.9 ± 1.6 mm (Table 2). The mean accuracy of all the measured sites was 6.3 ± 2.1 mm. Mean procedure time required for CSBMS (2.3 ± 0.9 versus 12.5 ± 4.6 seconds; *P* = 0.02) was less than the time required for Indian ink injection. All the patients received index and follow-up endoscopies with CSBMS with ease and without adverse events or technical failure. One CSBMS probe was used for all 50 procedures.

## 4. Discussion and Conclusion

In our pilot study, a novel CSBMS accurately identified previously marked gastric sites by endoscopic tattooing within 1 cm during follow-up endoscopy. No technical failure or adverse events associated with CSBMS were observed during index and follow-up endoscopy.

India ink is commonly used in the colon and esophagus for preoperative localization and surveillance of neoplastic lesions [13–15]. Inaccurate interpretation or inability to localize India ink marking at the time of surgery may occur in up to 15% of cases [14]. Long-term data on the durability of India ink beyond four years is lacking and has potential risk of fading [15]. Other noninvasive marking approaches have been developed but application in clinical settings has been limited [16–20]. Variation of anatomy and peristalsis among patients have also precluded the widespread use of non-India ink marking techniques in the stomach. Finally, submucosal tattoo injection may potentially interrupt the tissue planes and impact the feasibility of future mucosal resection. Therefore, we have developed a noninvasive, durable marking device that can be utilized during endoscopy for surveillance of gastric lesions.

In our initial work, IGBMS that utilizes a static gastric model identified previous tattoo site by 11 mm and 13 mm. Compared to the IGBMS, CSBMS uses a dynamic three-dimensional gastric model and also the number of reference points was increased from three to five. Furthermore, as part of the protocol, the stomach was maximally insufflated with air to reduce the variation in the size of the stomach between the index and follow-up endoscopies. In our study, the mean accuracy of the measured sites was improved to a mean distance of 6.3 mm. The accuracy of CSBMS observed in our study is likely acceptable for clinical practice given that the precision in traditional method is almost 6 mm (typical open biopsy forceps used in traditional examination span 6 mm). Given our preliminary results, CSBMS may provide an alternative method for marking premalignant gastric lesions to India ink injection. Furthermore, CSBMS may also serve a role for mucosal marking of colonic and esophageal lesions (i.e., colon polyps or dysplastic Barrett's esophagus). The entire CSBMS costs 8,000 US dollars, and the probe can be reused for multiple procedures. Finally, CSBMS was well tolerated by the patients without any adverse events and CSBMS was easily manipulated for a normal endoscopist even without extra training.

In summary, CSBMS accurately identified previously marked gastric sites by endoscopic tattooing within 1 cm during follow-up endoscopy. Future studies evaluating physiologic clinical endpoints in a large number of patients performed by multiple endoscopists are needed to validate the accuracy and effectiveness of CSBMS.

## Figures and Tables

**Table 1:** Clinical characteristic.

	*N* = 25
Median age, yr. (range)	57 (40–77)
Male	18 (72%)
Smoking	17 (68%)
Alcohol use	8 (32%)
Grading of intestinal metaplasia	
Mild IM	0
Moderate IM	4
Severe IM	21

SD: standard deviation.

IM: intestinal metaplasia.

**Table 2:** Accuracy and marking time.

Biopsy location	Biopsy accuracies, (mean's) mm	Marking time (mean's) s (injection/CSBMS)
Angularis	5.3 ± 2.2	12 ± 4.1/3 ± 1.8
Antral lesser curvature	5.7 ± 1.4	12 ± 4.4/2 ± 0.7
Antral greater curvature	6.1 ± 1.1	11 ± 3.6/2 ± 0.7
Anterior wall	6.9 ± 1.6	12 ± 5.5/2 ± 0.7
Posterior wall	6.9 ± 1.6	13 ± 5.2/2 ± 0.7
